# Label-Free Microfluidic Modulation Spectroscopy Monitors RNA Origami Structure and Stability

**DOI:** 10.3390/bios16030166

**Published:** 2026-03-16

**Authors:** Phoebe S. Tsoi, Lathan Lucas, Allan Chris M. Ferreon, Ewan K. S. McRae, Josephine C. Ferreon

**Affiliations:** 1 Department of Biochemistry and Molecular Pharmacology, Baylor College of Medicine, Houston, TX 77030, USA; 2 Center for RNA Therapeutics, Houston Methodist Research Institute, Houston, TX 77030, USA

**Keywords:** RNA origami, RNA folding, microfluidic modulation spectroscopy, infrared spectroscopy, kinetic trap

## Abstract

RNA origami enables genetically encoded, single-stranded RNA nanostructures that can self-assemble through co-transcriptional folding and are increasingly deployed as scaffolds for biosensing, synthetic biology, and nanomedicine. A recurring practical bottleneck is scalable, solution-phase readout of whether a designed scaffold has reached its intended base-paired architecture, whether it undergoes slow maturation or kinetic trapping, and how its stability is distributed across motifs. Here, we adapt microfluidic modulation spectroscopy (MMS) as a label-free structural biosensor for RNA folding by exploiting the rich 1760–1600 cm^−1^ vibrational fingerprints of RNA bases and base pairs. MMS alternates between sample and composition-matched buffer measurements in a microfluidic transmission cell to automatically subtract the solvent background, enabling high-quality spectral measurement from microliter volumes under native solution conditions. Using a six-helix-bundle-with-clasp (6HBC) RNA origami as a model, we established an analysis workflow (baselined second derivative and constrained deconvolution) to quantify paired versus unpaired populations. Thermal ramping resolves multiple unfolding events and yields an unfolding barcode that differs between young and mature ensembles. Importantly, MMS tracks post-transcriptional maturation from a kinetically trapped young conformer toward a more compact, base-paired mature state, consistent with prior cryo-EM/SAXS observations for 6HBC RNA origami. Together, these results position MMS as a rapid, automated, and scalable complement to high-resolution structure determination for engineering dynamic RNA origami biosensors.

## 1. Introduction

RNA molecules often populate heterogeneous folding intermediates with slow maturation steps, and kinetic traps that are challenging to monitor in solution [1,2,3,4]. These structural dynamics are increasingly being utilized in synthetic RNA devices to create biosensors, where conformational switching, motif positioning, and kinetic control determine signal fidelity [5,6]. RNA origami offers a genetically encoded route to large designer RNA nanostructures that fold co-transcriptionally from a single strand [7,8], and design frameworks such as the RNA Origami Automated Design (ROAD) platform [9] now enable rapid and systematic exploration of these architectures. Cryogenic electron microscopy (cryo-EM) and small-angle X-ray scattering (SAXS) of a six-helix-bundle-with-clasp (6HBC) RNA origami [10] revealed conformational heterogeneity and long-lived intermediates that resemble the topological misfolds of natural RNAs [11,12]. Individual particle tomography revealed a diverse ensemble of structures present at a single snapshot of 6HBC maturation [13], highlighting the need for solution-phase methods capable of resolving ensemble dynamics and RNA folding. 

These challenges highlight a practical need for biosensor development in synthetic RNA design and for studying RNA biology workflows: a solution-phase, label-free, scalable readout that reports folding completeness (paired versus frayed/unpaired populations), slow maturation and kinetic traps, and stability landscapes under environmental stressors (e.g., temperature, salts, ligands). High-resolution methods, such as cryo-EM, are transformative but remain relatively low throughput. Bulk methods such as gel electrophoresis can miss base pair class specificity or distort solution conformations. A vibrational spectroscopy readout that is intrinsic, quantitative, and compatible with native buffers would therefore fill a valuable gap for RNA nanodevice engineering and RNA structural characterization. 

Infrared (IR) spectroscopy is well-suited for this purpose because nucleobase vibrational modes (C=O, C=N, C=C, NH_2_ stretching, bending, and ring modes) are directly perturbed by hydrogen bonding, stacking, and local electrostatics. Classic compilations of nucleic acid vibrational bands established that the 1800–1500 cm^−1^ window contains a dense, information-rich set of base carbonyl stretches and ring vibrations whose frequencies and intensities shift with base pairing and conformation [14,15]. Recent community efforts, including the Nucleic Acid InfraRed spectroscopy DataBase (NAIRDB) of Fourier Transform Infrared spectroscopy (FTIR) spectra for nucleic acids, further emphasize that these base region signatures are highly sensitive to base pairing, sugar conformation, and backbone environment, while also highlighting the experimental challenge that liquid water absorbs strongly near ~1645 cm^−1^ and can mask nucleic acid features in conventional transmission FTIR experiments [16]. Time-resolved IR methods have also demonstrated that specific bands can be used as interaction-class reporters during RNA folding and unfolding, including temperature-jump IR measurements of RNA hairpins and tetraloops that resolve microsecond-to-millisecond pathways without external labels [17,18,19,20,21]. More recently, multidimensional IR approaches (2D IR) have provided complementary access to vibrational coupling and dynamical heterogeneity of nucleic acid bases and duplexes [22,23,24,25].

Despite this strong conceptual foundation, routine application of IR to RNA engineering is often limited by solvent background, pathlength constraints, and throughput. Standard transmission FTIR in aqueous solution frequently relies on D_2_O substitution to reduce interference in the base/amide region, while attenuated total reflectance FTIR (ATR-FTIR) introduces wavelength-dependent penetration-depth effects and can be sensitive to surface interactions and correction procedures [16]. These constraints are particularly acute for RNA origami, where screening multiple sequences and solution conditions may require rapid, automated, and low-volume measurements.

Microfluidic modulation spectroscopy–infrared (MMS) addresses these limitations by alternating the sample and a composition-matched reference buffer through a microfluidic transmission cell, enabling real-time rapid solvent/background subtraction and drift suppression. In protein biopharmaceutical characterization, MMS has been shown to generate spectra comparable to conventional FTIR while improving sensitivity, repeatability, and concentration range, without requiring D_2_O substitution in many workflows [26]. Quantitative studies have demonstrated that MMS can detect and quantify small fractions of misfolded/structural-impurity species at levels substantially below conventional FTIR limits of quantitation [27], and optimized acquisition parameters and instrument formats have been developed for robust, high-throughput secondary structure measurement in native formulations [28,29,30]. Importantly, for extending MMS beyond proteins, recent analytical work has begun applying microfluidic modulation approaches to RNA higher-order structure monitoring in solution [31].

Here, we extend MMS to RNA origami and demonstrate how MMS can function as a structural biosensor for RNA folding, maturation, and stability. Using the 6HBC RNA origami system as a case study, we implement similarity-spectrum processing and constrained deconvolution to quantify stably base-paired versus dynamically paired or unpaired spectral populations; resolve multi-step, base pair class-specific thermal unfolding signatures; and directly track hours-scale maturation from a young conformer into a mature, more base-paired ensemble, providing a rapid solution-phase complement to cryo-EM/SAXS-informed RNA origami design principles.

## 2. Materials and Methods

### 2.1. RNA Origami Design and Sample Preparation

The 6HBC sequence was transcribed as described previously [10]. A 0.5 mL transcription reaction was purified by size exclusion chromatography on a Superose 6 Increase 10/300 column (Cytiva, Marlborough, MA, USA) equilibrated with 25 mM HEPES pH 8.0, 50 mM KCl, and 5 mM MgCl_2_. The major peak was collected for downstream analysis. To generate conformer populations of varying maturation, RNA was incubated at room temperature and measured at defined time points corresponding to “young” (~30 min), “intermediate” (~4 h), and “mature” (~24 h) ensembles.

### 2.2. RedShift MMS Acquisition and Thermal Ramping

The Aurora TX (RedShiftBio Inc, Boxborough, MA, USA) is an automated MMS system that alternates RNA sample and a composition-matched buffer blank at 1 s intervals through a microfluidic transmission chamber for solvent subtraction, enabling spectra from microliter volumes in native buffers [26,27,28]. MMS uses PEEK tubing which is equilibrated prior to measurement to prevent non-specific sample binding. The flowcell contains a calcium fluoride window and nickel shims. Spectra were acquired across the RNA base region (~1760–1600 cm^−1^). Thermal ramping was performed from 25 to 95 °C at a ramp rate of 1 °C/min with spectra collected every 17 s. Minimum volume and concentration requirements are 75 µL at 0.1 mg/mL for single MMS experiments or 250 µL at 0.1 mg/mL for thermal experiments.

GQ RNA 

A measurement of 0.5 mg/mL GQ RNA (UUAGGG)_4_ was prepared in 10 mM Tris, 0.1 mM EDTA pH 7.4. A measurement of 100 µL of GQ RNA with or without 10 mM KCl was additionally buffer exchanged using ZebaSpin desalting columns (Thermo Fisher Scientific, Waltham, MA, USA) to ensure proper buffer matching before loading onto a 96-well microplate (Thermo Fisher Scientific).

polyA RNA

A measurement of 0.5 mg/mL polyA RNA (Sigma-Aldrich, St. Louis, MO, USA) was buffer exchanged to 25 mM HEPES pH 8.0, 50 mM KCl. MMS and thermal ramp experiments were conducted.

6HBC RNA Origami

A measurement of 0.5 mg/mL 6HBC RNA was buffer exchanged to 25 mM HEPES pH 8.0, 50 mM KCl, and 5 mM MgCl_2_. MMS and thermal ramp experiments were conducted at 0, 4, and 24 h timepoints.

### 2.3. Spectral Processing, Similarity Spectra, and Constrained Deconvolution

Raw solvent-subtracted absorbance spectra were baseline-corrected and smoothed using Savitzky–Golay filtering [32]. Overlapping bands were resolved by computing an inverted second derivative (“similarity spectrum”), S(ν) = −d^2^A(ν)/dν^2^, which enhances subtle absorbance features and suppresses broad backgrounds. Peak deconvolution was performed by fitting multiple constrained components with centers initialized from reference spectra of mononucleotides and model base pairs. Deconvolved components were grouped into base pair-assigned versus unpaired/base ring-assigned classes and integrated to estimate the fractional base-paired population (%BP) versus unpaired/free population (%Unpaired) for each condition. Predicted (labeled as Predicted* in figures) base-pairing percentage of RNA origami was calculated from ROAD-derived secondary structures, assuming that kissing-loop motifs, UUCG loops, and junction regions are more dynamic than canonical duplex RNA, and therefore contribute weakly or not at all to the IR-detected base-paired population.

## 3. Results

### 3.1. Base and Base Pair Spectral Library Enables RNA Folding Readouts by MMS

**Figure 1:**
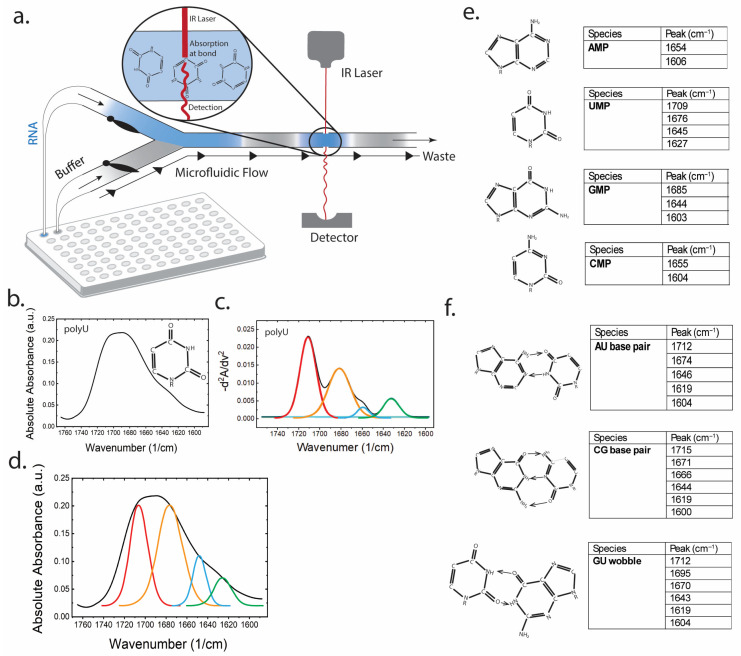
Microfluidic modulation IR (MMS) enables assignment of RNA nucleobase and base pair marker bands in the 1760–1600 cm^−1^ window. (**a**) Schematic of MMS measurement: Alternating RNA sample and matched buffer segments flow through a microfluidic transmission cell intersecting a mid-IR laser beam; rapid modulation enables real-time solvent/background subtraction and drift suppression. (**b**) Representative absolute absorbance spectrum for polyU. (**c**) Inverted second-derivative (“similarity”) spectrum of polyU resolving overlapping bands attributable to uracil with Gaussian curves overlaid at: 1710 (red), 1676 (orange), 1645 (blue), and 1627 (green) cm^−1^). (**d**) Example peak model/deconvolution of the polyU absorbance envelope into component bands corresponding to the uracil vibrational sub-modes. The same color-coded Gaussian curves, albeit scaled, from panel c are overlaid over the absolute spectra. (**e**) Summary table of characteristic peaks for AMP, UMP, GMP, and CMP used as single-nucleotide references for assigning spectral features to base functional groups. (**f**) Summary table of characteristic peaks for AU, CG, and GU wobble base pairs linking peak positions to functional group vibrations that report on hydrogen bonding and base pair identity.

Figure 1 establishes the mapping between chemical structure and mid-IR fingerprints for RNA bases and base pairs within the 1760–1600 cm^−1^ window [33]. Mononucleotide reference spectra provide anchor points for base-specific carbonyl and ring modes, while model base pair spectra (AU, CG, and GU wobble) reveal pairing-induced shifts and splitting patterns that serve as markers of hydrogen bonding environments. These reference markers motivate a pattern-based interpretation framework.

### 3.2. Potassium-Induced Folding of G-Quadruplex RNA Validates MMS as a Solution-Phase RNA Folding Sensor 

To establish that MMS reports authentic RNA folding transitions driven by solution conditions, we examined the potassium-dependent folding of a canonical RNA G-quadruplex (GQ RNA) formed by the sequence (UUAGGG)_4_. RNA G-quadruplexes are well-characterized noncanonical RNA structures stabilized by monovalent cations, particularly K^+^, which coordinate within the central channel formed by stacked G-quartets and promote Hoogsteen hydrogen bonding and base stacking. Extensive structural and biophysical studies have shown that telomeric RNA sequences adopt stable, predominantly parallel-stranded G-quadruplex topologies in K^+^ solution, whereas folding is significantly weakened or disrupted in the absence of stabilizing cations [34,35,36]. 

**Figure 2:**
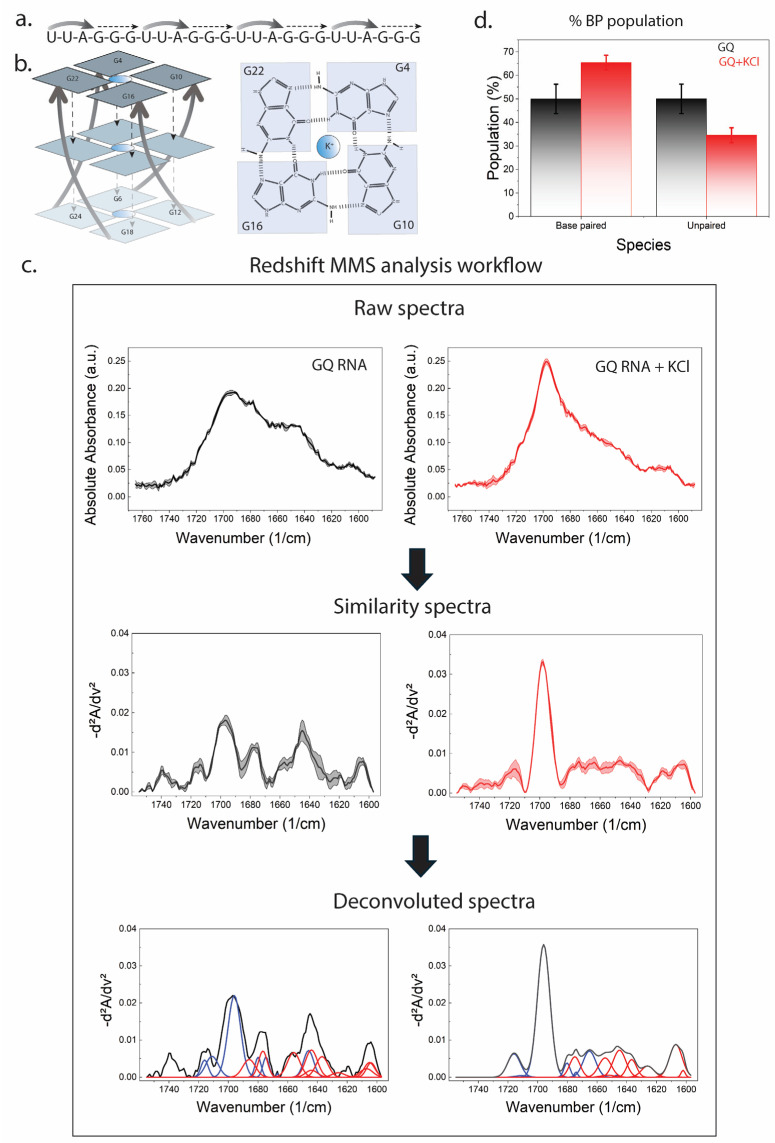
Potassium ion-induced folding of a G-quadruplex RNA. (**a**) Primary sequence of the G-quadruplex-forming RNA, (UUAGGG)_4_, highlighting the guanine-rich repeats that enable formation of stacked G-quartets. (**b**) Structural schematics of the folded RNA G-quadruplex. Left: Cartoon representation of stacked G-quartets forming a parallel quadruplex architecture. Right: Atomic-level depiction of a single G-quartet, illustrating Hoogsteen hydrogen bonding between guanines and coordination of a central K^+^ ion, which stabilizes the quadruplex stack. (**c**) MMS analysis workflow applied to GQ RNA in the absence (black) and presence (red) of KCl. Top: Solvent-subtracted raw absorbance spectra in the RNA nucleobase region (1760–1600 cm^−1^). Middle: Inverted second derivative (“similarity”) spectra (-d^2^A/dν^2^), which enhance overlapping vibrational features associated with guanine carbonyl and ring modes. Bottom: Constrained peak deconvolution of similarity spectra into component bands corresponding to paired/stacked guanine interactions (blue/gray) and unpaired or non-quadruplex-associated contributions (red). (**d**) Quantification of base-paired (%BP) versus unpaired spectral populations derived from integrated deconvolved components for GQ RNA in the absence and presence of KCl. Addition of potassium ions increases the base-paired population and reduces unpaired contributions, consistent with ion-induced stabilization of G-quadruplex folding. Error bars represent mean ± SEM.

MMS spectra of GQ RNA collected in the absence of KCl display broad, overlapping features in the 1760–1600 cm^−1^ nucleobase region, consistent with a heterogeneous ensemble dominated by unpaired or weakly interacting guanine bases (Figure 2c). Upon addition of KCl, pronounced spectral redistribution is observed, including sharpening and intensity increases in guanine-associated carbonyl and ring-mode bands. These changes are consistent with enhanced base stacking and Hoogsteen hydrogen bonding associated with G-quartet formation. Similar K^+^-dependent stabilization and spectroscopic signatures of RNA G-quadruplex folding have been reported using NMR, circular dichroism, UV melting, and mass spectrometry [34,35,37]. 

Processing of MMS absorbance data into similarity spectra and constrained deconvolution reveals a marked increase in base-paired spectral components upon KCl addition, accompanied by a corresponding decrease in unpaired contributions (Figure 2c,d). Quantification of integrated deconvolved components shows that potassium shifts the ensemble toward a substantially higher base-paired population, directly reporting the folding transition at the ensemble level. This behavior is consistent with prior experimental and computational work demonstrating that RNA G-quadruplex folding proceeds through a multi-pathway landscape in which cation binding stabilizes stacked G-quartets relative to unfolded, hairpin, or triplex intermediates [38]. Together, these results validate MMS as a general, solution-phase method for monitoring RNA folding transitions induced by environmental variables such as ionic composition.

### 3.3. MMS Fingerprints Quantify Global Base Pairing in 6HBC RNA Origami

**Figure 3:**
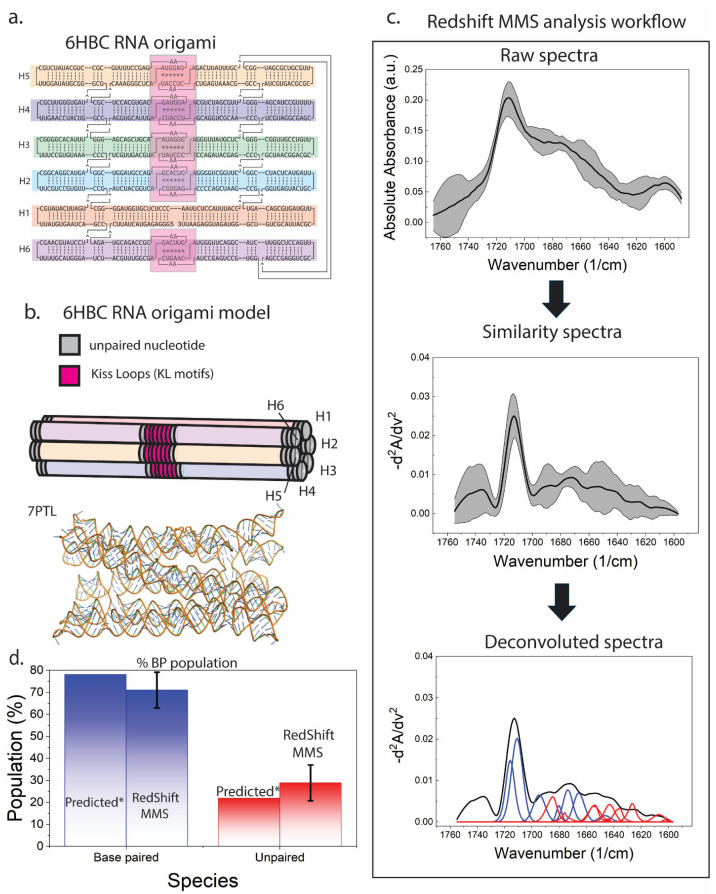
RNA origami target and MMS workflow for quantifying stably base-paired vs. dynamic or unpaired populations. (**a**) Secondary structure schematic of the 6-helix-bundle-with-clasp (6HBC) RNA origami, with helices and motifs color-coded. (**b**) Tertiary structure model illustrating the 6HBC bundle architecture and the placement of kissing-loop (KL) motifs; structural reference shown for the mature conformer (PDB: 7PTL [39]). (**c**) MMS analysis pipeline: Raw absorbance spectra (mean ± SEM), conversion to similarity spectra via inverted second derivative processing, and peak deconvolution into base pair (blue) and unpaired (red) components. (**d**) Base pair population estimated from the integrated deconvolved components compared against design-predicted (Predicted*) base-paired vs. free nucleotide fractions. Design-predicted (Predicted*) base-paired and unpaired populations were calculated based on the assumption of dynamic helix termini, junctions, and tertiary motifs.

The 6HBC RNA origami is a co-transcriptionally folded scaffold comprising 10 four-way junctions, five internal kissing loops, 40 A-form helical regions, and 12 UUCG tetraloops. Together, these elements stack to form six contiguous bundles (Figure 3a,b). This highly paired design was previously shown to undergo a complex structural rearrangement that necessitates melting of double helical elements to allow for maturation into its compacted form [10,13]. The MMS workflow (Figure 3c) converts raw absorbance spectra into baselined second derivative spectra and then into deconvolved peak components, enabling quantitative separation of base pair-assigned signals from unpaired. From integrated deconvolution areas, the mature 6HBC ensemble is estimated at ~70% base-paired and ~30% free/unpaired, compared with a design-predicted ~78% base-paired and ~22% free (Figure 3d). The predicted (labeled as Predicted*) base-paired and free percentages were calculated assuming that KL motifs, helix termini, junctions, and tertiary motifs in RNA origami remain dynamic [10] and can contribute fraying and transiently disrupted pairs that appear as an “unpaired” spectral population, especially early in the maturation process.

### 3.4. Thermal Ramping Resolves Multiple Base Pair Class Unfolding Events in Mature 6HBC

**Figure 4:**
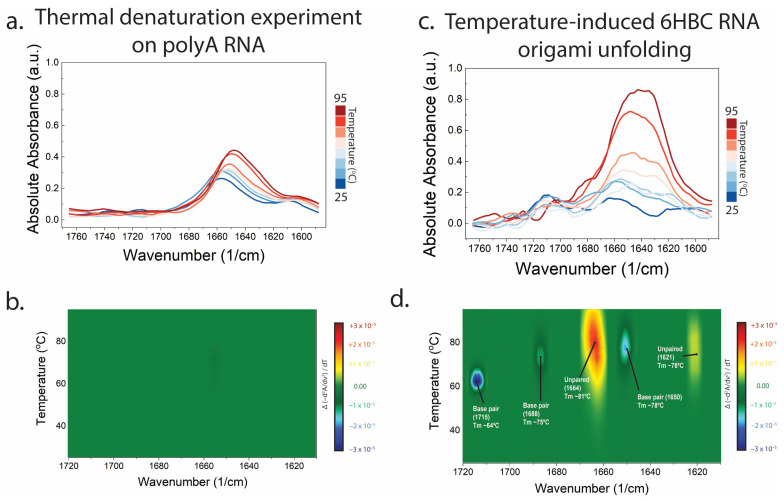
Temperature-induced unfolding of polyA RNA and 6HBC RNA origami resolved by MMS. (**a**,**c**) Raw MMS absorbance spectra collected during a thermal ramp (25–95 °C), showing temperature-dependent redistribution of intensity across the nucleobase carbonyl/ring-mode region. (**b**,**d**) Heat map of temperature-dependent similarity signal highlighting distinct transitions. Annotated midpoints correspond to apparent transition temperatures for each feature.

Thermal ramping reveals that unfolding is multi-step rather than a single cooperative melt (Figure 4). We first establish that thermal features are not observed in a non-folding polyA RNA sample (Figure 4a,b). For mature 6HBC, the temperature-wavenumber similarity map displays distinct transitions at ~1715 cm^−1^ with an apparent midpoint near 64 °C, at ~1688 cm^−1^ near 75 °C, at ~1650 cm^−1^ near 78 °C, and in features associated with unpaired bases (e.g., ~1664 cm^−1^ for a band near 81 °C and ~1621 cm^−1^ for a band near 78 °C). Together these features form an unfolding “barcode” that reports how stability is distributed across AU-rich, GC-rich, and frayed/unpaired elements of the structure.

### 3.5. MMS Directly Tracks Hours-Scale Maturation from a Young Conformer

**Figure 5:**
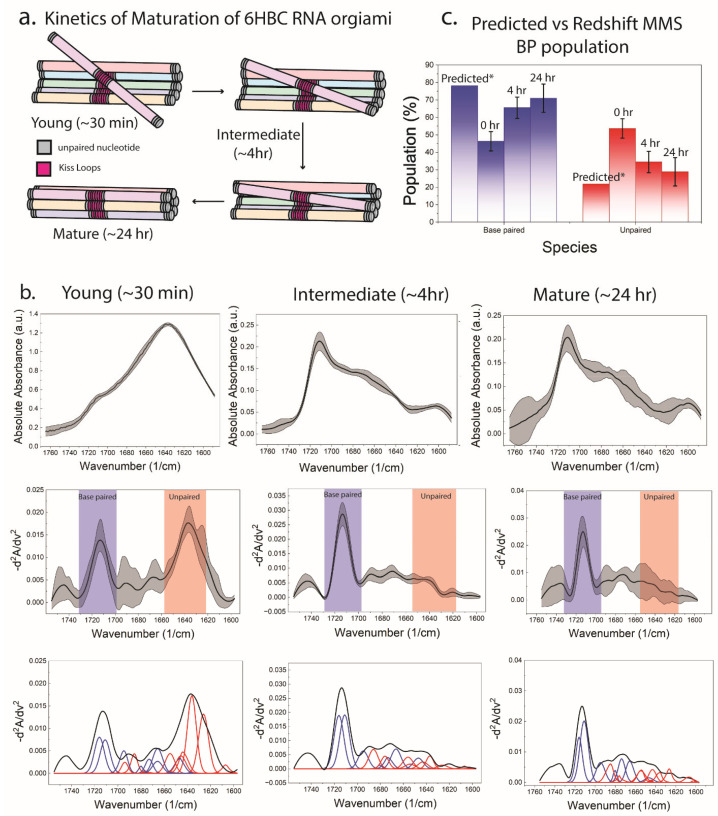
Time-dependent maturation of 6HBC RNA origami tracked by MMS. (**a**) Conceptual model of post-transcriptional maturation from a “young” open conformer (~30 min) through an intermediate (~4 h) to a “mature” closed conformer (~24 h), consistent with slow tertiary rearrangements in RNA origami. (**b**) For each time point, raw absorbance (mean ± SEM, **top**), similarity spectra (mean ± SEM, **middle**; base-paired region shaded blue, unpaired region shaded orange), and deconvolved peak components (**bottom**) show progressive gain of base pair-assigned contributions (blue Gaussian fits) and loss of unpaired contributions (red Gaussian fits). (**c**) Quantified base-paired vs. unpaired population across maturation time points compared to design-predicted values (mean ± SEM). Predicted* base-paired and unpaired populations were calculated based on the assumption of dynamic KL motifs.

A core contribution of the RNA origami cryo-EM/SAXS study was the identification of late structural maturation in 6HBC due to a kinetic folding trap released after hours [10]. RedShift MMS provides a complementary solution-phase readout of this maturation in a straightforward time course (Figure 5). Quantification by deconvolution shows a shift toward base pairing: the young ensemble (~30 min) is ~46% base-paired/~54% unpaired, the intermediate (~4 h) ensemble is ~65% base-paired/~34% unpaired, and the mature (~24 h) ensemble is ~71% base-paired/~29% unpaired (Figure 5c). This provides a quantitative readout of ensemble changes (slow folding completion and trap escape) that can be monitored across different constructs, buffers, and additives with good throughput.

### 3.6. Young vs. Mature Ensembles Show Distinct Thermal Fingerprints Consistent with Different Folding Landscapes

**Figure 6:**
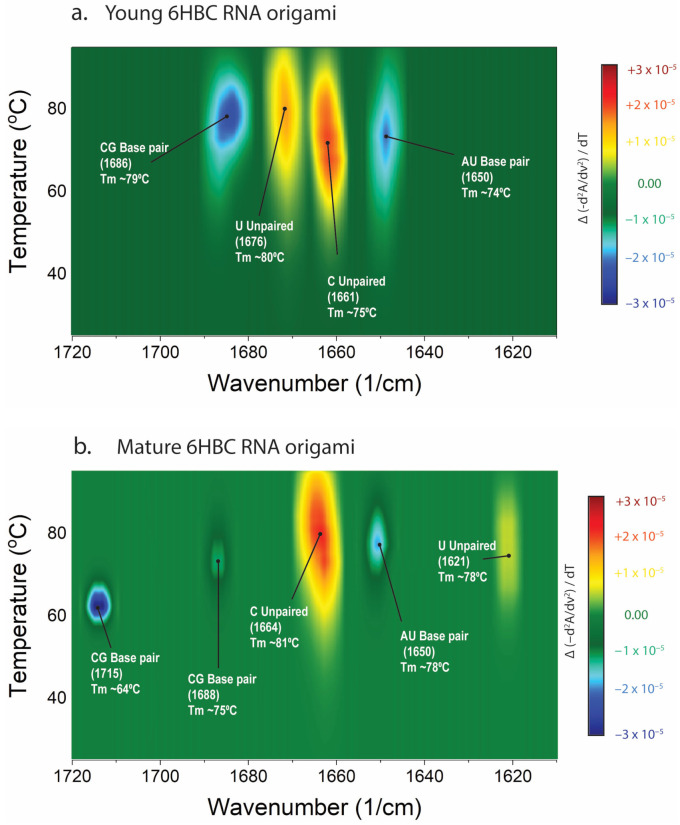
Thermal-derivative fingerprints reveal distinct unfolding pathways in young vs. mature 6HBC RNA origami. (**a**) Heat map (Δ similarity signal/ΔT) for young 6HBC showing dominant high-temperature transitions (e.g., CG feature near 1686 cm^−1^ and AU features near 1650 cm^−1^) with annotated apparent midpoints. (**b**) Equivalent map for mature 6HBC showing additional lower-temperature CG-associated transition near 1715 cm^−1^ alongside higher-temperature transitions, indicating maturation-dependent redistribution of stability across motif classes.

Maturation changes not only the overall paired fraction, but also which interaction classes melt at specific temperatures (Figure 6). In young 6HBC, transitions are dominated by a CG-associated loss of signal near 1686 cm^−1^ at ~79 °C, an unpaired uracil signal increase near 1676 cm^−1^ at ~80 °C, an unpaired cytosine near 1661 cm^−1^ at ~75 °C, and AU-associated signal decrease ~1650 cm^−1^ at ~74–75 °C. In contrast, mature 6HBC exhibits a barcode that includes a prominent lower-temperature loss of base-pairing signal at ~1715 cm^−1^ at ~64 °C (absent in young), alongside higher temperature CG and AU loss of signal (e.g., ~1688 cm^−1^ near 75 °C and ~1650 cm^−1^ near 78 °C). The emergence of the ~1715 cm^−1^ event in the mature ensemble suggests that maturation produces a stronger canonical base-pairing signal compared to the young RNA.

## 4. Discussion

### 4.1. IR Signatures of RNA Structural Transitions and Consistency with the Nucleic Acid IR Literature

RNA folding changes hydrogen bonding, base stacking, and local electrostatics, which redistribute intensities and shift frequencies among nucleobase carbonyl stretches and ring modes. These effects are a well-established basis for interpreting nucleic acid FTIR spectra: compilations of base region bands emphasize that base pairing generally perturbs carbonyl stretches most strongly, whereas ring and mixed modes provide complementary sensitivity to stacking and conformation [14,15]. Community curation of nucleic acid spectra similarly highlights the base region as particularly informative for conformational changes, while noting the practical challenges imposed by aqueous backgrounds [16]. In this context, our MMS spectra and similarity spectra behave as expected for a large RNA nanostructure: CG-associated carbonyl features (near 1715 and 1688 cm^−1^), AU-associated contributions (near 1650 cm^−1^), and unpaired base/ring features (e.g., ~1664 and ~1621 cm^−1^) evolve as partially independent reporters rather than collapsing into a single two-state melting transition.

### 4.2. Relationship of MMS RNA Spectra to Standard FTIR/ATR-FTIR and to Time-Resolved IR Studies of RNA Folding

MMS is a linear mid-IR absorption measurement, so the underlying band positions and assignments are expected to align with conventional FTIR/ATR-FTIR when referenced to the same solvent and temperature conditions. Accordingly, the wavenumber regions we use overlap with classic RNA folding reporters used in time-resolved IR: for example, temperature-jump IR studies have probed bands near 1620 cm^−1^ (largely reporting A/U interactions) and 1661 cm^−1^ (largely reporting G/C interactions) to resolve multiphasic RNA folding kinetics and incorrectly folded intermediate populations [17,18]. IR thermodynamic studies of UNCG tetraloops and related stem-loop motifs likewise exploit base carbonyl and ring-mode markers to disentangle loop versus stem contributions and to quantify sequence-dependent stability [19,20,21]. Our MMS thermal-derivative/similarity maps are conceptually similar to these different IR approaches in that they emphasize small, temperature-dependent redistributions of overlapping bands.

### 4.3. Advantages of MMS over Conventional IR for RNA Origami Workflows

Routine IR of nucleic acids in water is often constrained by solvent absorption and experimental geometry. NAIRDB notes that water has a strong vibration around ~1645 cm^−1^ that can mask nucleic acid signals in the base region, motivating D_2_O substitution in transmission measurements and careful consideration of ATR corrections and artifacts [16]. MMS directly addresses these constraints by utilizing a mid-IR laser for increased optical power and by modulating RNA and composition-matched buffer measurements to reduce noise and signal drift with microliter-scale consumption [26]. Although much of the peer-reviewed MMS validation has focused on protein higher-order structures, the performance characteristics are directly relevant to RNA: MMS can provide FTIR-comparable spectra with improved repeatability at low concentrations [26,28], can detect small structural impurities/misfolded fractions at quantitation limits well below conventional transmission FTIR [27,30], and can be integrated with automated thermal ramping to generate stability fingerprints [29,40]. Recent application of microfluidic modulation approaches to RNA higher-order structure monitoring further supports the feasibility of extending this platform to nucleic acid analytics [31]. Together, these advantages align well with RNA origami development needs, where screening of sequence variants, folding conditions, and device environments benefit from rapid, quantitative, solution-phase readouts.

### 4.4. Mapping MMS Observables onto the 6HBC Kinetic Trap/Maturation Model and Implications for RNA Origami Biosensors

McRae et al. propose that 6HBC experiences a kinetic folding trap involving kissing-loop partner connectivity and topological constraints during bundle compaction, with trap release only after ~10 h and additional slow compaction thereafter [10]. Using solution SAXS to resolve folding/maturation intermediates in co-transcriptional RNA origami, they observed a “young” conformer that relaxed to a more compact “mature” state on ~10–12 h timescales after transcription. Time-dependent scattering changes consistent with an internal structural rearrangement were observed. Importantly, the intermediate SAXS curves were quantitatively captured as evolving mixtures of the two endpoint conformers (guided by cryo-EM models), with additional gradual compaction before and after the main transition. This orthogonal, solution-phase detection of intermediate conformational populations by SAXS supports our interpretation of the MMS-IR measurements as reporting on transient intermediate states during maturation rather than a single, concerted folding event. Our MMS time series reproduces the key phenomenology in a label-free solution assay: the young ensemble exhibits elevated unpaired signatures and a simplified thermal barcode, while maturation increases the paired fraction and yields a richer, redistributed unfolding fingerprint. In practical biosensor terms, a scaffold can appear “present” in structural assays yet remain spectroscopically immature, with frayed or misregistered regions that compromise motif positioning and switching behavior. MMS provides a quantitative readout of folding completeness that can complement cryo-EM’s high-resolution snapshots and SAXS’s global compaction measures by offering time-/temperature-resolved reporting in solution.

### 4.5. Broader Context: IR Observables for RNA Dynamics and Future Opportunities

Beyond equilibrium thermal melts, the wide literature demonstrates that IR spectroscopy can resolve dynamic RNA processes without extrinsic labels, including microsecond folding kinetics initiated by laser temperature jumps [17,18], sequence-dependent melting/refolding pathways in tetraloop motifs [20,21], and the extraction of thermodynamic contributions from distinct structural elements [19]. At the same time, multidimensional vibrational methods (2D IR) have revealed anharmonicity and coupling among nucleobase vibrations and how duplex melting reshapes structural and solvation dynamics [22,23,24,25]. While these ultrafast approaches are distinct from MMS, they suggest a roadmap for richer mechanistic interpretations of the MMS fingerprints: isotope editing or targeted motif engineering could help disentangle overlapping base and backbone contributions, and comparisons to curated datasets (e.g., NAIRDB) could support more systematic spectral-to-structure mapping [16]. In RNA origami specifically, MMS could be applied to screen designs for reduced kinetic trapping (faster rise in %BP and disappearance of young-like barcodes), to identify buffer conditions that accelerate maturation, and to quantify stability margins under deployment conditions in a manner compatible with iterative biosensor optimization.

### 4.6. Limitations and Interpretive Considerations

Several caveats accompany the present interpretation. First, nucleic acid IR bands are highly coupled and rarely map one-to-one onto a single structural feature, so the most reliable readout is the coordinated evolution of multiple bands and deconvolved populations [14,15,16].

Second, in complex RNAs, spectral changes can reflect not only Watson–Crick pairing but also stacking, fraying, protonation, and local electrostatic changes that alter transition dipoles and coupling patterns. In our measurements, 6HBC evolves from ~45% to ~70% IR-assigned “paired” populations during maturation. At face value, a large fraction of apparently unpaired RNA is difficult to reconcile with cryo-EM models of the young conformer, in which the helical elements are already well formed and resolvable. This suggests that the MMS-derived pairing metric does not report simply on topological pairing, but instead reflects the fraction of bases engaged in near-ideal, stably stacked A-form geometry on the IR timescale.

In the pre-maturation ensemble, base pairs may be present but distorted by shearing, stretching, buckling, propeller twist, or transient opening as the structure relaxes and reorganizes. Such deviations from ideal geometry are expected to weaken or redistribute carbonyl and ring-mode signatures and thereby reduce the apparent paired population in IR measurements. Even in the mature structure, dynamic regions such as junctions, kissing loops, and tetraloop motifs are likely to sample non-optimal geometries more frequently than extended duplex RNA, further reducing their contribution to the IR-visible paired signal. From this perspective, the remaining discrepancy between structure-based expectations and MMS-derived populations is plausibly explained by persistent local distortions and dynamics rather than by extensive true unpairing.

Together, these considerations support interpreting the MMS-derived percentages as a measure of stable, IR–visible base pairing rather than absolute pairing stoichiometry. Future experiments that correlate MMS signatures with motif-specific perturbations or higher-resolution probes of base pair geometry will be important for disentangling the respective contributions of pairing, stacking, and distortion to the observed spectra.

Third, because MMS relies on matched-buffer subtraction, careful buffer matching (including salts, co-solutes, and temperature-dependent baselines) remains essential. Finally, the constrained deconvolution approach improves interpretability but introduces model dependence; future work can refine reference libraries (including motif-specific references) and validate assignments by orthogonal perturbations.

## 5. Conclusions

MMS provides a label-free, solution-phase platform that translates RNA nucleobase vibrational fingerprints into quantitative readouts of folding and dynamics. Using 6HBC RNA origami, MMS quantifies paired versus unpaired populations in folded ensembles, resolves multi-step, base pair class-specific thermal unfolding signatures, and tracks slow maturation consistent with kinetic trap behavior previously reported for RNA origami. These capabilities support scalable, screening-oriented design cycles for RNA origami biosensors and dynamic RNA nanodevices.

## Data Availability

Dataset available upon request from the authors.

## Abbreviations

The following abbreviations are used in this manuscript:

MMS	Microfluidic Modulation Spectroscopy
6HBC	Six-Helix-Bundle-with-Clasp
ROAD	RNA Origami Automated Design
Cryo-EM	Cryogenic Electron Microscopy
SAXS	Small-Angle X-ray Scattering
NAIRDB	Nucleic Acid InfraRed Spectroscopy Database
FTIR	Fourier Transform Infrared Spectroscopy
ATR-FTIR	Attenuated Total Reflectance Fourier Transform Infrared Spectroscopy
KL	Kissing Loop
